# The Acute Effects of Standing on Executive Functioning in Vocational Education and Training Students: The Phit2Learn Study

**DOI:** 10.3389/fpsyg.2022.810007

**Published:** 2022-03-17

**Authors:** Petra J. Luteijn, Inge S. M. van der Wurff, Amika S. Singh, Hans H. C. M. Savelberg, Renate H. M. de Groot

**Affiliations:** ^1^Faculty of Educational Sciences, Open University of the Netherlands, Heerlen, Netherlands; ^2^Mulier Institute, Utrecht, Netherlands; ^3^Department of Nutritional and Movement Sciences, NUTRIM, School of Nutrition and Translational Research in Metabolism and SHE, School of Health Professions Education, Maastricht University, Maastricht, Netherlands

**Keywords:** sit-to-stand desk, executive functioning, cognitive functioning, sedentary behavior, vocational education and training students, sitting

## Abstract

Research suggests that sedentary behavior (SB) is negatively associated with cognitive outcomes. Interrupting prolonged sitting has been shown to improve cognitive functions, including executive functioning (EF), which is important for academic performance. No research has been conducted on the effect of standing on EF in VET students, who make up a large proportion of the adolescent population and who are known to sit more than other students of this age. In this study, we investigated the acute effects of reducing SB by short time standing on EF in vocational education and training (VET) students. In a randomized crossover study, 165 VET students were first taught for 15 min in seated position. After this, they performed while seated the Letter Memory Test for updating, and the Color Shape Test for shifting and inhibition. Students were randomly assigned to a sitting or standing condition. All students were taught again for 15 min and then took the same tests in the condition they were allocated to, respectively, standing or seated. After 1 week, the test procedure was repeated, in which students switched conditions. Mixed model analyses showed no significant effect of sitting or standing on updating, shifting, or inhibition. Also, no significant differences were found for the order of condition on updating, shifting, or inhibition. Our results suggest that 40 min of standing does not significantly influence EF among VET students.

## Introduction

Students generally spend a large part of their school day seated (Kariippanon et al., 2019). However, it is well known that sedentary behavior (SB) such as sitting, and especially prolonged periods of SB without interruption, is negatively associated with physical and mental health outcomes (Tremblay et al., 2011; Suchert et al., 2015; Biddle et al., 2019; van der Berg et al., 2019). It has also been suggested that breaking up SB positively affects executive functioning (EF; Mullane et al., 2017; Rosenbaum et al., 2017; Mazzoli et al., 2019). Furthermore, it has been shown that light intensity physical activity (PA), including standing is beneficial for several markers of insulin sensitivity and plasma lipids, and that light intensity PA, such as standing, has a greater health effect than one-time intensive PA (Duvivier et al., 2013, 2017, 2018). Since standing during class is more feasible to implement than other forms of PA, for example, sports into the school day, the aim of the current study was to investigate the effect of standing during class on the EF in vocational education and training (VET) students, who are known to sit more than other students of this age (Bernaards, 2013; van Engen and Christoffels, 2017). Considering the biological mechanisms that have been shown to occur during standing, standing is hypothesized to have small positive effects on the EFs updating, inhibition and shifting compared to sitting.

The term EF refers to a family of top-down mental processes (Diamond, 2013) and to the abilities needed for metacognitive control and direction of mental experience (Lezak et al., 2004). EF is needed to concentrate and to pay attention (Diamond, 2013) and is important for academic performance (Pluck et al., 2019; Wang and Zhou, 2019; Dubuc et al., 2020). EF consists of capacities that enable a person to engage successfully in independent, purposive, self-directed, and self-serving behavior (Lezak et al., 2004). EF involves one’s ability to actively maintain task goals and goal-related information and use this information to effectively bias lower-level processing (Miyake and Friedman, 2012). In general, three core EFs are distinguished as: (a) updating, (b) shifting, and (c) inhibition (Miyake et al., 2000). Updating is the act of modifying the current status of a representation of schema in memory to accommodate new input, and entails monitoring and encoding incoming information and appropriately revising the items in working memory (WM), by replacing no longer relevant information with new, more relevant information (Morris and Jones, 1990). Shifting involves moving back and forth between multiple tasks, operations, or mental sets (Monsell, 1996, as cited in St Clair-Thompson and Gathercole, 2006). It is not that demanding to keep doing what you have been doing, but shifting, i.e., shifting back and forth between mental sets, is one of the most demanding EFs (Diamond, 2013). Task switching improves during child development and declines during aging (Diamond, 2013). Inhibition involves the control over stimuli irrelevant to task performance (interference control) and the inhibition of habitual responses (Stroop, 1935). Inhibition enables us to selectively attend, focusing on what we choose and to suppress attention to irrelevant stimuli and involves the discipline to stay on task despite distractions and completing a task despite temptations to give up, to move to more interesting work, or to have a good time instead (Diamond, 2013). EF is strongly and positively associated with school performance and academic success (Christopher et al., 2012; Gray et al., 2015; Samuels et al., 2016). This means that EF covers a broad spectrum of cognitive skills and that fostering EF is important for school performance.

The acute effects of PA, including standing, on cognitive performance and EF seem to be caused by several biological mechanisms, including increased blood circulation in the brain (Weijenberg et al., 2011), improved insulin sensitivity, lower fasting plasma triacylglycerols levels (Duvivier et al., 2013, 2017), and improved sensitization for glucose transport across the blood–brain barrier (Chandrasekaran et al., 2021). These mechanisms are positively correlated to EF (Reay et al., 2006; van den Berg et al., 2009; Gonzales et al., 2010; Guiney et al., 2015; Sliz et al., 2020; Chandrasekaran et al., 2021). Additionally, standing leads to increased heart rate (Ebara et al., 2008), indicating increased arousal (Ebara et al., 2008; Knight and Baer, 2014). Arousal has shown to be associated with attention (Byun et al., 2014).

The effect of standing on EF has been investigated in several studies, for example, in the study of Rosenbaum et al. (2017). In this study, university students stood while executing a 72 item Stroop test measuring inhibition. The students in the standing condition performed better on this test than the students in the sitting condition (Rosenbaum et al., 2017). Additionally, studies with standing interventions of longer duration than conducted in the study of Rosenbaum and colleagues also reported positive effects of standing on EF. For example, one single “reduced sitting” school day, meaning that adolescents sat for 50% less time than during a “normal” school day (i.e., a normal school day consists of 240 min of sitting time) and with no bouts of sitting >20 min, resulted in improvements in mental attention capacity in adolescents (12–15y) at the end of the school day (Penning et al., 2017). Additionally, Mazzoli et al. (2019) demonstrated a weak but significant correlation between more sit-to-stand transitions over two school days and both enhanced attention and improved reaction time in an inhibition test in children (6–8 y; Mazzoli et al., 2019). Similarly, Mullane et al. (2017) found significant improvements in WM and attention in overweight adults who stood for 10, 15, 20, and 30 min, respectively, throughout the day for four consecutive weeks, compared to the control condition, in which participants sat for 4 weeks. However, in the same study, no effect was found of 10, 15, 20, and 30 min standing during the day on shifting. Furthermore, Bantoft et al. (2016) showed that standing up to 60 min compared to sitting did not lead to significant effects on short-time memory, WM, and attention in young adults (22.67 y; Bantoft et al., 2016). Also, Schwartz and colleagues found no significant differences in attention and inhibition performance in students (20–32 y) when comparing alternating sitting and standing, and sitting-only during two assessment days (Schwartz et al., 2018).

In most school systems, the amount of SB increases as children and adolescents progress through the school years. In other words, the average sitting time and the proportion of prolonged sitting without interruption are higher among older students than among younger students, which can partially be explained by the reduced proportion of class time spent on PA among the higher age groups than among the lower age groups (Mooses et al., 2017). Also evidence was found for a clear relationship between school environment and students’ PA and SB, namely, through the availability of sit-to-stand desks, the encouragement by the school to exercise, and the teacher’s attitude toward PA (Morton et al., 2016). Additionally, an association between students’ academic schedule and both SB and PA has been shown (Chim et al., 2020). Thus, interrupting sitting behavior is particularly important among older adolescents.

In summary, results from previous studies on the effect of standing in the classroom on EF are mixed and therefore, no conclusion can be drawn about the effect of acute standing on EF compared to sitting. However, considering the biological mechanisms of standing, a small positive effect can be expected of short-time standing on EF in VET students. Many of the studies had relatively small sample sizes and focused mainly on young children. However, no research has been conducted on the effect of standing on EF in VET students, who make up a large proportion of the adolescent population (CBS, 2013), and who are known to sit more than other students of this age (Bernaards, 2013; van Engen and Christoffels, 2017). Furthermore, VET students are a very diverse group of students which show great differences in the mastery of basic knowledge and skills. They generally find self-regulation of learning difficult, as reflection on learning outcomes and learning strategy use is often limited (van Engen and Christoffels, 2017). In general, they score lower on (digital) problem solving than peers attending university of applied sciences or university, which is strongly related to the application of learning strategies and the level of language and numeracy skills (Christoffels and Steehouder, 2015). Therefore, we aim to investigate the acute effect of standing at sit-to-stand desk versus sitting behind a traditional desk during class on acute EF in VET students.

## Materials and Methods

### Design

The current study is part of the PHIT2LEARN project (PHysical activity InTerventions to enhance LEARNing). Overall, the goal of PHIT2LEARN was to investigate the effects of PA/SB interventions on a variety of outcome measures in VET students. In nine different sub-studies (i.e., the current study concerns only one sub-study), physical activity behavior interventions of VET students and its effects on their cognitive performance and mental wellbeing were examined. Among other things, short-term intervention studies were designed to investigate the acute effect of exercise interventions and breaking up sitting on learning performance measures of VET students. Furthermore, the students’ perceptions of the implementation of sit-to-stand desks and what they believe are needed to encourage students to stand more during class. Two of the studies have been published (Golsteijn et al., 2021; Kirschner et al., 2021). The current study was a randomized-controlled trial with a crossover design. Students stood behind sit-to-stand desks or remained seated as a control condition. Ethical approval was obtained from the Research Ethics Committee (cETO) of the Open University (reference U2017/00519/FRO) and the study has been registered in the Dutch Trial Register (NTR6358).

### Participants

A total of 219 VET students were invited from 12 classes from the study tracks Child care, Youth care, and Teaching assistant from the levels 2, 3, and 4 of a VET institution in the south of Netherlands. VET is the most practical level of the Dutch tertiary education (CBS, 2013). VET is subdivided into four levels and prepares students for executive jobs or middle management jobs (i.e., dependent on the level). There were no exclusion criteria. All students received oral and written information about the research during an information session. Students were given at least 1 week to consider their participation, after which, if they agreed to participate, they signed an informed consent form.

Initially, we wanted to run an RM ANOVA for the statistical analysis. According to the power analysis conducted for this purpose, using an effect size of 0.15 and a power of 0.8 (Penning et al., 2017), a sample of 128 participants would be sufficient. To achieve a power of 0.95, 196 participants would be sufficient. However, advancing insight made us decide to use mixed model analysis to investigate our main research question. Therefore, the power analysis appropriate for mixed model analysis was performed post-hoc. To do so, a power analysis was conducted by performing a Monte-Carlo simulation study with 1,000 simulated datasets per analysis, using the package simr (Green and Macleod, 2016). Since no suitable literature was found for updating and inhibition with a similar study design, the power calculation was executed based only on previous literature for shifting (Miyake et al., 2004; Graham et al., 2021). For shifting, a difference of 13.75 in decrease in reaction time of the correct answers between the pre-measurement and the post-measurement was specified between the intervention condition and the control condition (i.e., the intervention condition had 13.75 ms more decrease in reaction time of the correct answers in the post-measurement than in the pre-measurement compared to the control condition), calculated from the data obtained from the literature. Thus, our post-hoc power analysis showed that to obtain a power of 0.80 or more, a sample size of at least 920 participants was needed.

### Materials

In this study, we applied two well-validated neuropsychological tests that cover the three major domains of executive functioning (i.e., updating, shifting, and inhibition) and have been shown in previous research to have high test–retest reliability in previous research (Paap and Sawi, 2016; Soveri et al., 2018). Both tests were conducted on laptops with a 15.75 inch (i.e., 40 cm) screen, provided by the researchers.

#### Letter Memory Test

The Letter Memory Test (LMT) measures the updating capacity of the WM (Inman et al., 1998). Participants were presented with a series of five, seven, or nine consonant letters without repeat, one at a time. At the end of each series of letters, the participant had to recall the last three presented letters by clicking these in a provided letter matrix with all possible letters presented. Participants did not have to display the letters in the correct order. When a participant did not remember the last three letters, it was possible to click “blanco” for the letters he/she did not remember. Participants completed three training trials first, followed by twelve test trials. At the beginning of a trial, a fixation cross was displayed for 1,000 milliseconds (ms) in the center of the screen. Each stimulus was displayed for 2,500 ms in the center of the screen. The total number of correctly recalled letters on the LMT was used as a measure of WM/updating. The maximum score that could be obtained was 36 points (i.e., 12 trials with three letters per trial to remember). The median sensitivity and specificity of the LMT have been found to be 0.943 and 1.0, respectively (Inman et al., 1998). The internal reliability was.61 in previous research (Friedman et al., 2008).

#### Color Shape Test

The Color Shape Test (CST) measures both shifting and inhibition (Miyake et al., 2004; Miyake and Friedman, 2012). The test started with 16 training trials followed by 64 test trials. In each round (i.e., consisting of 64 trials with the same command), participants were presented with a shape (i.e., a triangle or a circle), a colored block (i.e., a red or green block), or a combination of both (i.e., a red triangle, a green triangle, a red circle, or a green circle). For the shape round, participants had to answer whether they saw a circle or a triangle, and for the color round, whether they saw a red block or a green block by pressing the “A” for “red” or “circle” and the “L” for “green” or “triangle.” In the first round, shapes (i.e., shape test) were presented; in the second round, colors (color test) were presented. In the third round (i.e., shifting test), shape and colors were presented alternately. The presentation of shapes and colors was at random; in some cases, a task was repeated (i.e., two color trials or two shape trials after each other), and in other cases, the participants needed to switch between the shape task and the color task (i.e., shifting; a color trial after a shape trial or the other way around). In the fourth (i.e., shape inhibition-task) and fifth round (i.e., color-inhibition-task), shapes superimposed on color patches were presented (i.e., a circle superimposed on a red square). Participants had to indicate the shape (i.e., round four) or color (i.e., round five), regardless of the underlying color or superimposing shape, respectively. The interval between response and presentation of next stimulus was 600 ms; the size of a stimulus was 36 mm. The stimulus was presented until the participant had responded. In all rounds, the outcome measure was the mean response time (RT) of the correct answers. Shift costs are the differences in RT of the correct answers between switching trials in round three and the RT of the correct answers in round one and two. Inhibition costs are the differences in RT of the correct answers between inhibition trials in round four and five and the RT of the correct answers in round one and two. In previous research, the reliability was 0.86 (Friedman et al., 2008), whereas the test–retest correlation was 0.75 in previous research (Paap and Sawi, 2016).

#### Additional Measures

All participants filled in a questionnaire regarding sex, age, and school class, as there are indications that there is a relationship between sex, age, and EF (Jacobsen et al., 2017; Grissom and Reyes, 2019).

### Procedure

**Figure 1:**
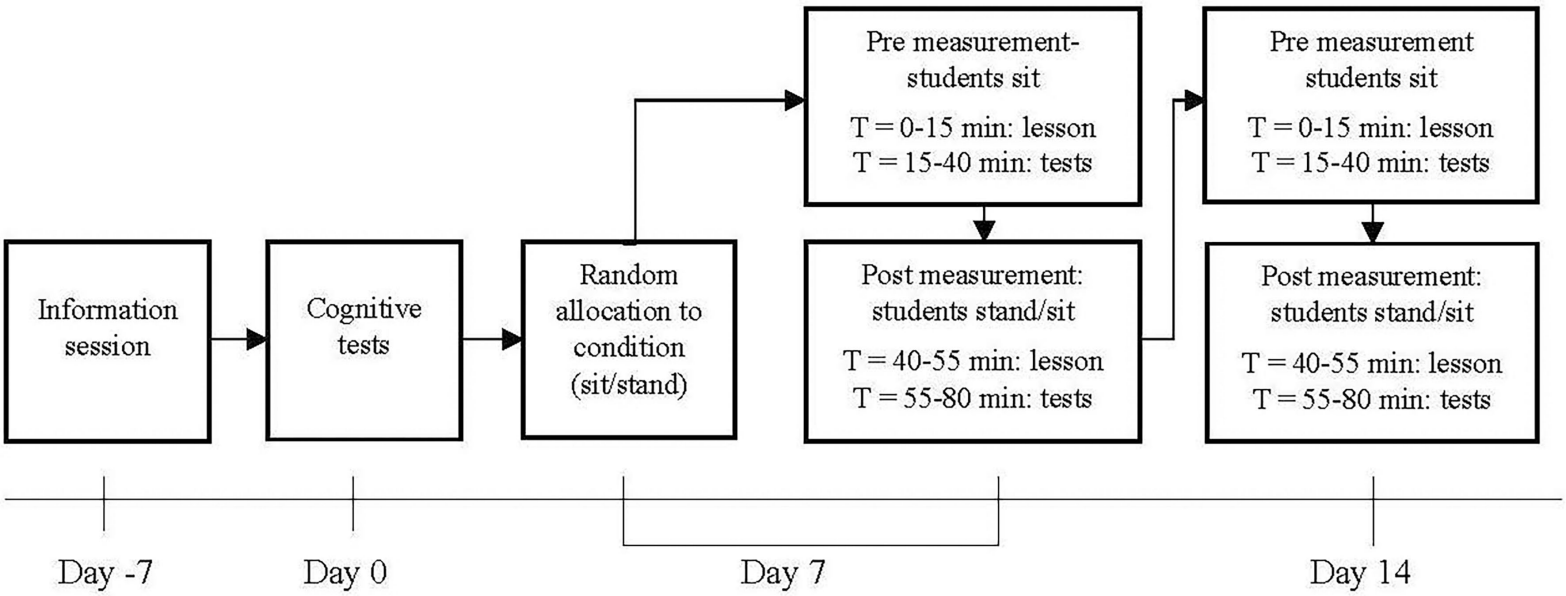
Schematic representation of the study design. Students who sat in the first test session at day 7, stood in the second test session at day 14 and vice versa.

After the consent of the management and the selection of the participating classes, an information session for the teachers was organized. During this session, the teachers were given oral and written information about the study and were told what was expected of them. Then, during an information session, students were given oral and written information about the study and received an informed consent form. They were given 1 week to consider their participation. After that week, a practice session took place in which participants performed the cognitive tests while seated, to familiarize them with the tests. At the beginning of the practice session, students received an explanation of the tests. After another week, the first test session took place in a classroom at the participants’ school, which contained both sit-to-stand desks and traditional desks. Students were randomized into a sitting or standing condition as follows: since dual sit-to-stand desks were used in this study, students were “paired” with a fellow student of the same height; the two tallest individuals formed a pair, the next two students, and so on. These pairs were then randomized into two groups (e.g., a standing and a sitting group) using an online randomizer. After this, the researchers placed a card with the assigned condition, a form with the names of the students who would be sitting at that desk, and for each student a laptop from the research team and a word search with pencil on each desk. Students were then asked to take their seats at the desks with the form with their names on it. When the students were seated, the teacher taught for 15 min about citizenship, during which time all participants remained seated. After this 15-min lesson, participants first filled out the background questions, then performed the LMT and the CST, which took approximately 25 min. Participants who finished the tests early could solve the provided word search. After all participants finished the tests, the students who were assigned to the standing group raised their desks and stood up. When the students assigned to the standing condition were standing, the teacher taught for another 15 min, after which the participants again performed the LMT and CST as a post-measurement immediately after the lesson. Here, the students who were in the standing condition remained standing and the seated students remained seated. This procedure was repeated 1 week later, with participants switching test conditions during the second part of the test session (i.e., those who stood during test session 1 sat during test session 2 and vice versa). Participation in the study was voluntary, but only students who had participated in the practice session could participate in the test sessions. Students who did not give informed consent, or who had not participated in the practice session, were given a “normal” lesson that took place in a separate room or in the back of the classroom. Figure 1 shows the schematic representation of the study design.

### Data Processing

For the LMT, the score per participant per test session and test time (i.e., pre-test or post-test) were calculated. For the CST, the blocks with less than 32 correct answers were removed as it is expected that in that case the participants did not understand the test. Afterward, in the shifting round (i.e., the third round), all non-shifting trials were removed (i.e., a color trial after a color trial or a shape trial after a shape trial). Then, all congruent trials were removed in the inhibition rounds (i.e., the fourth and fifth round, requiring the same key to be pressed for shape and color). Subsequently, all incorrect answers and all latencies of <170 ms and  > 5,000 ms were excluded as it is assumed that if participants react too quickly, they have not been able to think, and if they react too slowly, they have been distracted (Sternberg, 1999; Miyake et al., 2004; Harrison et al., 2010). Finally, the data were structured in such a way that per participant one line remained with all mean response times for shifting and for inhibition per test session and per test moment. Shift costs were calculated by subtracting the average RT in rounds one and two from the average RT in round three, which was the shifting task. Trials that were not switching tasks (i.e., two consecutive shape or color trials) were excluded from this calculation. Inhibition costs were calculated by subtracting the mean RT in rounds one and two from the mean RT in rounds four and five, which are the inhibition tasks, excluding all congruent trials in the inhibition tasks. Congruent trials are trials in which the stimuli are in agreement with each other (i.e., a triangle on a green patch or a circle on a red patch), require the same response, and thus do not measure inhibition.

### Statistical Analyses

Analyses were conducted in SPSS (version 24; SPSS Inc., Chicago, Il, United States) with the significance level set at 0.05. To assess the effects of the sitting or standing condition on the outcomes for updating, shifting, and inhibition, controlling for three covariates (i.e., sex, age, and school class), mixed model analyses were performed. First, it was checked whether mixed model analyses were necessary, this was done by comparing the −2 log likelihoods (−2LL) of a model with a fixed intercept for participants and a model with a random intercept for participants and both condition and order of condition (i.e., first stand then sit or reverse) as independent variable. After these two analyses, models were built in a stepwise way. Separate models were built for each outcome measure (i.e., updating, shifting, and inhibition scores). One covariate was added and the new model was compared to the previous model. If the change in -2LL indicated a significant improvement of the model, the variable was kept in the model. Variables were added in the following order: (I) class code as third level, (II) pre-test scores, (III) test session (i.e., test day), (IV) age, and (V) sex.

## Results

### Participants’ Characteristics

**Figure 2:**
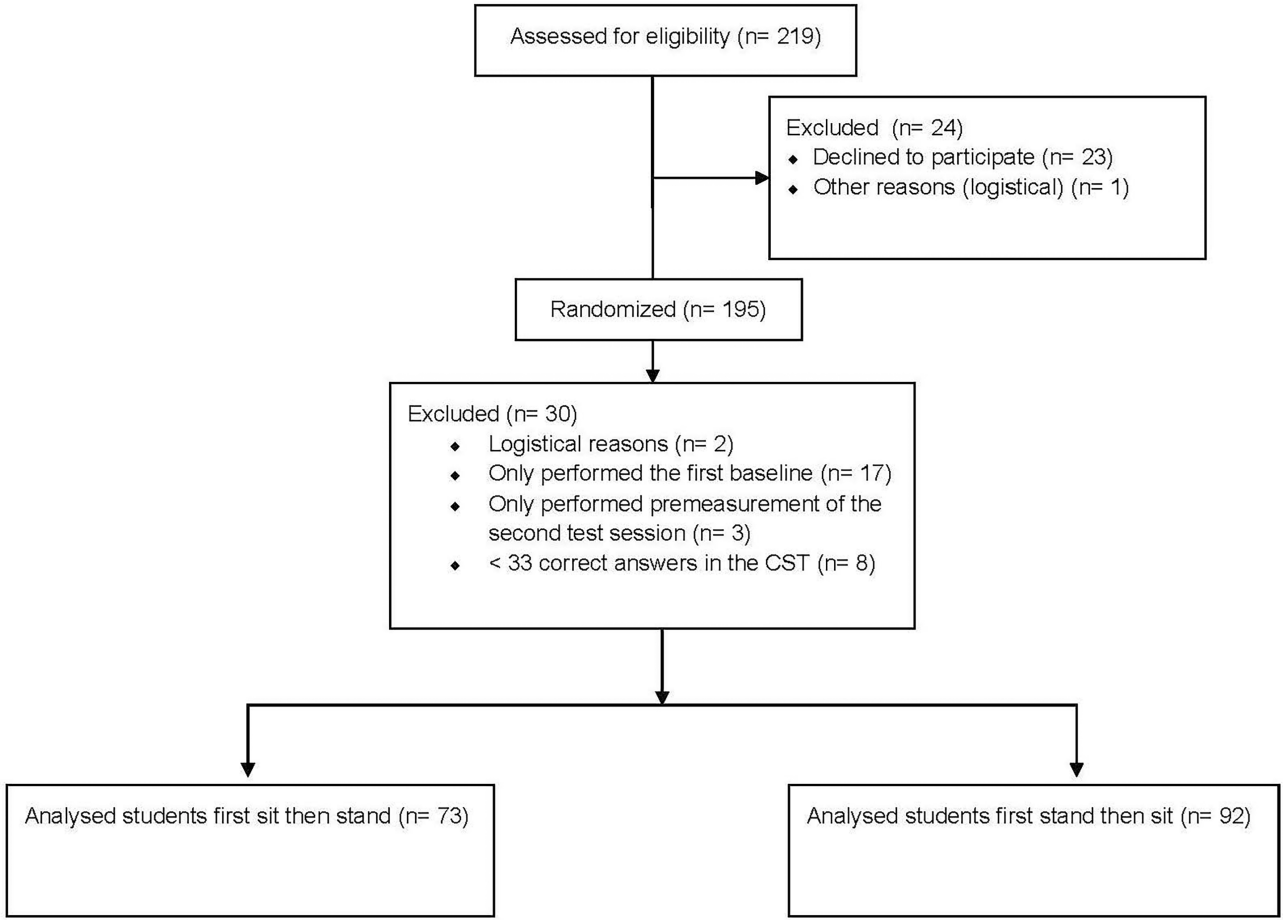
Flow diagram: progress of participants through the trial.

A total of 219 students were invited for this study. Of these, 23 students did not want to participate, resulting in 196 students which we included in the study. During the trajectory, participants dropped out due to the following reasons: logistical reasons (*N* = 1), class/group allocation unclear (*N* = 2), no show up during the test sessions (*N* = 20), and one or more blocks with <33 correctly answered trials in CST (*N* = 8). Additionally, a total of 4 and 20 trials, respectively, were excluded as the latency was <170 ms and > 5,000 ms, respectively. Note that no participants were excluded nor were any trial within the latencies indicated above. Ultimately, results of 165 students (106 boys, 18.8 y, SD = 7.9, Table 1) were included in the data analyses. A flow diagram including the numbers and reasons for exclusion can be found in Figure 2.

**Table 1:** Participants’ characteristics (*N* = 165) total and per order of condition.

		Total	Sit-stand	Stand-sit
Age [M (SD)]	Missing	18.8(7.9)	18.1(1.3)	19.32(10.6)
	1	0	1
Sex (N)	M	106	44	62
	F	59	29	30
	Missing	0	0	0

*M*, *Mean; SD*, *Standard deviation; Sit-stand: sitting followed by standing; and Stand-sit: standing followed by sitting*.

### Executive Functioning and the Impact of Standing

No significant effect of sitting or standing (*B* = 0.51, SEb = 0.50, 95%CI = [−0.49, 1.51], Table 2) or order of these conditions (*B* = −0.34, SEb = 0.60, 95%CI = [−1.54, 0.85]) on updating scores was found. When comparing the mixed models for the effect of standing on updating, the best model was the model with random intercept, with participant as a second level, class code as a third level, and updating pre-measurement and age as covariates. The only covariate that was significant was updating pre-measurement (*B* = 0.86, SEb = 0.58, 95% CI = [0.75, 0.97], Table 2), indicating that students who scored better at the pre-test, also scored better at the post-tests. Table 3 shows an overview of the mean and standard deviation (SD) of scores of the cognitive test.

No significant effect of sitting or standing (*B* = −9.47, SEb = 15.05, 95%CI = [−39.30, 20.36], Table 2) or condition order (*B* = −9.57, SEb = 26.39, 95%CI = [−61.67, 42.54], Table 2) on shifting scores was shown. When comparing the mixed models for the effect of standing on shifting, the best model was the model with random intercept and participant as a second level and shifting pre-measurement and age as covariates. The only covariate that was significant was shifting pre-measurement (*B* = 0.14, SEb = 0.07, 95% CI = [0.01, 0.28], Table 2), indicating that students who scored better at the pre-test, also scored better at the post-tests.

**Table 2:** Results of mixed models analyses for effect of standing on updating, shifting, and inhibition scores.

**Updating**	***B***	**SE b**	**95% CI**
*-2LL 1546.875 df 8*			
Intercept	2.77	1.94	[−1.06, 6.60]
Condition standing	0.51	0.50	[−0.49, 1.51]
Condition order	−0.34	0.60	[−1.54, 0.85]
Updating pre-measurement	0.86	0.58	**[0.75, 0.97]**
Age	0.03	0.04	[−0.04, 0.10]
**Shifting**			
*-2LL 3223.504 df 7*			
Intercept	89.03	36.29	**[17.37, 160.68]**
Condition standing	−9.47	15.05	[−39.30, 20.36]
Condition order	−9.57	26.39	[−61.67, 42.54]
Shifting pre-measurement	0.14	0.07	**[0.01, 0.28]**
Age	1.29	1.52	[−1.72, 4.31]
**Inhibition**			
*-2LL 2678.058 df 6*			
Intercept	42.60	17.38	**[8.20, 77.00]**
Condition standing	0.70	11.20	[−21.51, 22.90]
Condition order	6.61	14.28	[−21.61, 34.83]
Age	0.23	0.75	[−1.27, 1.72]

-2LL, −2Log Likelihood; df, degrees of freedom; *B* = Value; SEb, Standard error; CI. Confidence interval; Significant 95% CI scores are depicted in bold; condition = standing compared to sitting; and condition order = first sit then stand or vice versa.

**Table 3:** Mean scores of the cognitive tests.

	Sitting pre	Sitting post	Standing pre	Standing post
Updating [M, (SD)]	31.4(5.3)	30.2(6.6)	31.5(5.0)	30.8(6.1)
Shifting [M, (SD)]	163.3(153.6)	135.4(173.1)	157.4(132.5)	120.5(140.0)
Inhibition [M, (SD)]	33.4(79.2)	51.6(92.5)	49.3(90.7)	52.6(92.1)

*M, Mean; SD, Standard deviation*.

No significant effect of sitting or standing (*B* = 0.70, SEb = 11.20, 95%CI = [−21.51, 22.90], Table 2) or condition order (*B* = 6.61, SEb = 14.28, 95%CI = [−21.61, 34.83], Table 2) on inhibition scores was shown. When comparing the mixed models for the effect of standing on inhibition, the best model was the model with random intercept and participant as a second level and age as a covariate. Our analyses revealed that results did not differ with regard to age, gender, and school class.

## Discussion

The aim of the current study was to investigate the acute effect of standing at sit-to-stand desk versus sitting behind a traditional desk during class on EF in VET students. It was hypothesized that standing for 40 min would have small positive effects on the EFs updating, inhibition, and shifting. Since the results of our study suggest that 40 min of standing did not significantly change EF among VET students, the hypothesis was not supported.

In line with our results, previous research showed that standing for, respectively, 60 min a day (Bantoft et al., 2016), or regular sit-to-stand transitions during 2 days (Schwartz et al., 2018), or, respectively, 10, 15, 20, and 30 min standing throughout the day for four consecutive weeks (Mullane et al., 2017) had no effects on shifting, short-time memory, WM, selective and sustained attention, information processing speed, and inhibition. In contrast, in the study of Rosenbaum et al. (2017), where students stood while executing a 72 item Stroop test measuring inhibition, improvements in attention were found (Rosenbaum et al., 2017). Furthermore, a 50% reduced school day (Penning et al., 2017), sit-to-stand transitions during 2 days (Mazzoli et al., 2019), and successively 10, 15, 20, and 30 min standing during the whole day (Mullane et al., 2017) also lead to improved attention. As these studies mainly focused on young children or adults (Bantoft et al., 2016; Mullane et al., 2017; Penning et al., 2017; Rosenbaum et al., 2017; Schwartz et al., 2018), the results of those studies may not be generalizable to the population in the current study. An explanation for the fact that we found no effect of short-term standing on the EF of VET students may be that any neurocognitive benefits of standing, responsible for improvements in EF (Duvivier et al., 2013, 2017), might only become apparent after an extended period of time, as the neurological changes as mentioned in the introduction possibly only appear after a longer duration of standing than conducted in the current study. Another explanation may be that the students experienced the CST, which consisted of 5 rounds of testing with 64 stimuli each, as too long. It is thus possible that the length of this test may has affected the results.

The current study has some strengths. One strength is the crossover design, which has the advantage that all participants act as their own control. Furthermore, a crossover design eliminates confounding effects attributable to the characteristics of a specific group (Parienti and Kuss, 2007). Additionally, a strength of the current study is the use of a practice session to reduce the learning effect (Beglinger et al., 2005).

Our study has also some limitations. First, the study was underpowered. Originally, we intended to analyze the study results with a repeated measure ANOVA, but based on advancing insights, we decided that mixed model analysis would be more appropriate since this analysis takes the variance within a participant and the school class in which the participants are in into account. Therefore, we performed a post-hoc power analysis to determine the number of participants needed for this study when utilizing mixed model analysis. This post-hoc power analysis showed that 920 participants were needed to obtain sufficient statistical power. This means that the current study is underpowered, which may have negative consequences for the reliability of the outcomes. However, the current study included many more participants than previous studies in this domain, herewith still adding to the existing knowledge base around the role of standing education for EF. As the study was underpowered for the mixed model analysis executed, we executed additional mixed ANOVA analyses, mixed ANOVA is an extension of RM ANOVA for analyses with multiple independent variables, and the results of these analyses did not differ from the mixed model analysis (see the results in Supplementary Table S1) indicating that the results of the mixed model analysis are reliable.

Based on this study’s findings, it can be concluded that standing for 40 min does not affect EF in VET students. The results of the current study may be of interest to educational practitioners. Since it is shown that standing once for 40 min does not have an effect on the EF of VET students, it is not useful to let them stand for that duration. However, since previous research has shown that more standing and less sitting are beneficial to health (Duvivier et al., 2013, 2017, 2018), and standing is practical within a classroom and not detrimental to classroom behavior or learning (Sherry et al., 2016), it is recommended that sitting behavior of VET students is reduced by allowing them to stand. Future research could be conducted with enough participants and with a longer standing intervention than applied in the current study. Additionally, the effect of a more intensive PA intervention than short-time standing on EF can be investigated, whereby it is important that the intervention can be realistically implemented in a VET classroom.

## Data Availability Statement

Publicly available datasets were analyzed in this study. This data can be found here: https://doi.org/10.17026/dans-zmd-jzma.

## Ethics Statement

The studies involving human participants were reviewed and approved by Research Ethics Committee (cETO) of the Open University (reference U2017/00519/FRO) and the study has been registered in the Dutch Trial Register (NTR6358). Written informed consent to participate in this study was provided by the participants’ legal guardian/next of kin.

## Author Contributions

RG was responsible for the study design and the funding of the whole Phit2Learn project. PL and IW analyzed the data. PL wrote the first draft of the manuscript, which has been further revised by all the authors. All authors contributed to the article and approved the submitted version.

## Funding

The PHIT2LEARN study was funded by the Nationaal Regieorgaan Onderwijs (Netherlands Initiative for Education Research) by grant number 405 16 412.

## Conflict of Interest

The authors declare that the research was conducted in the absence of any commercial or financial relationships that could be construed as a potential conflict of interest.

## Publisher’s Note

All claims expressed in this article are solely those of the authors and do not necessarily represent those of their affiliated organizations, or those of the publisher, the editors and the reviewers. Any product that may be evaluated in this article, or claim that may be made by its manufacturer, is not guaranteed or endorsed by the publisher.
