# Supplementary material for: The Acute Effects of Standing on Executive Functioning in Vocational Education and Training Students: The Phit2Learn Study
Source: Front Psychol. 2022 Mar 17;13:810007. doi: 10.3389/fpsyg.2022.810007 (PMC8968320; doi:10.3389/fpsyg.2022.810007)
Supplement: Supplementary file 1 [file Table_1.pdf]

Supplementary file

Table S1 | Mixed *ANOVA of the effect of condition on the outcome measures*

	**Pre-measurement (M, SD)**	**Post-measurement (M, SD)**	**Difference pre-post measurement (M, SD)**
**Updating**			
F(1, 97) = 1.7, p=.2, η^2 =.02			
Sit-group	31.4, 5.3	30.2, 6.6	1.2, 1.3
Stand-group	31.5, 5.0	30.8, 6.1	0.7, 1.1
**Shifting**			
F(1, 86) = 3.5, p=.06, η^2 =.04			
Sit-group	163.3, 153.6	135.4, 173.1	27.9, 19.5
Stand-group	157.4, 132.5	120.5, 140.0	36.9, 7.5
**Inhibition**			
F(1, 70) = 1.3, p=.7, η^2=.002			
Sit-group	33.4, 79.2	51.6, 92.5	18.2, 13.3
Stand-group	49.5, 90.7	-1.3, 4.9	50.8, 85.8

Note that this statistics is comparing the standing to the sitting condition.
